# Supplementary material for: Recent increases in tropical cyclone rapid intensification events in global offshore regions
Source: Nat Commun. 2023 Aug 24;14:5167. doi: 10.1038/s41467-023-40605-2 (PMC10449825; doi:10.1038/s41467-023-40605-2)
Supplement: Supplementary file 1 — Supplementary Information [file 41467_2023_40605_MOESM1_ESM.pdf]

Supporting Information for "Recent Increases in Tropical Cyclone Rapid Intensification Events in Global Offshore Regions"

Yi Li[1,2], Youmin Tang[1,3]*, Shuai Wang[4], Ralf Toumi[5], Xiangzhou Song[1,2], Qiang Wang[1,2]

[1] College of Oceanography, Hohai University, Nanjing, China

[2] Key Laboratory of Marine Hazards Forecasting, Ministry of Natural Resources, Hohai University, Nanjing, China

[3] University of Northern British Columbia, Prince George, Canada

[4] Department of Geography and Spatial Sciences, University of Delaware, Newark, DE 19716, USA

[5] Department of Physics, Imperial College London, London, SW7 2AZ, UK

*Corresponding author: Youmin Tang, ytang@unbc.ca

Content:

Supplementary Note 1.

Supplementary Fig. 1 to 19.

Supplementary References

Supplementary Note 1.

We implement an improved Ordinary Least-Square (OLS) algorithm[1] because the robustness of linear regression is usually prone to the choice of period. This OLS algorithm assumes a time series can be decomposed in the following form,

$$y(t) = Bt + \sum A_i \cdot \sin(\omega_i t + \varphi_i) + N_t, \qquad (1)$$

where B is the true secular linear trend coefficient. $\sum A_i \cdot \sin(\omega_i t + \varphi_i)$ denotes multiscale internal variations, with A_i, ω_i and φ_i being the amplitude, frequency, and phase of *i*th oscillatory term, respectively. N_t represents noises. As derived by Lian (2017)[1], the linear trend of Equation (1) is

$$r(L) \cong B - \frac{6}{L^2} \sum \frac{A_i}{\omega_i} [\cos(\omega_i L + \varphi_i) + \cos(\varphi_i)], \qquad (2)$$

which consists of two components, one representing the true trend and one representing the uncertainty caused by internal oscillations. As the magnitude of the sum of the terms in the bracket in Equation (2) cannot be greater than 2, we have

$$\left||r(L) - B|\right| \leq B_{th} = \frac{12}{L^2} \sum \frac{A_i}{\omega_i}, \qquad (3)$$

where B_{th} is the theoretical threshold. For a given amplitude, low-frequency variations (small ω_i) will have a greater impact on the estimated trend than high-frequency variations (large ω_i). The secular linear trend coefficient B has the upper and lower limits of $r(L) - B_{th} \leq B \leq r(L) + B_{th}$. Then one can calculate if the sign of B would be the same as $r(L)$ by comparing the amplitudes of $r(L)$and B_{th}.

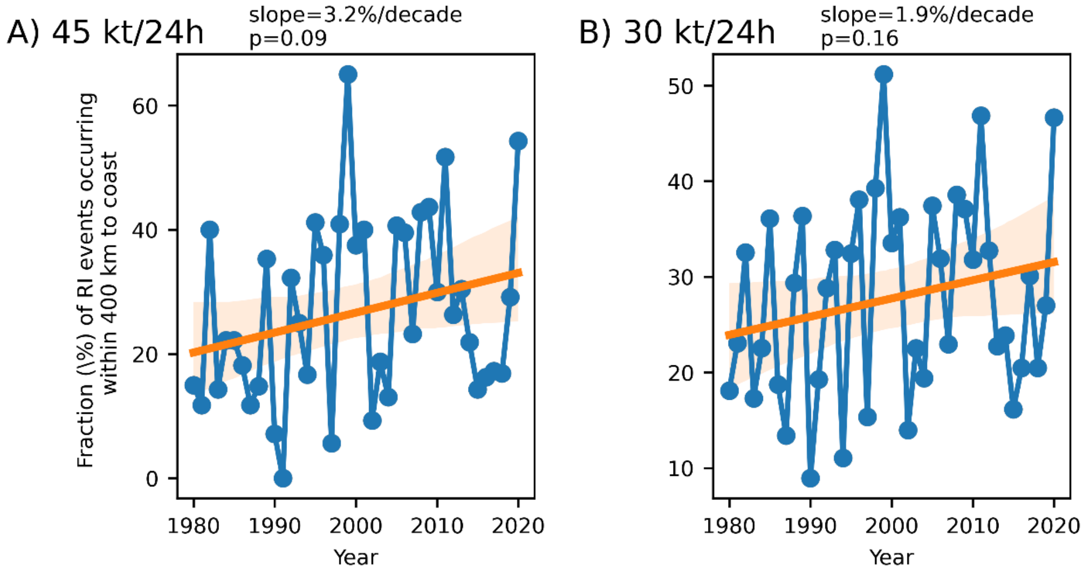

Supplementary Figure 1. Trends of fractions of annual TC activity within 400 km to land. Fraction is calculated as the ratio between RI events over the offshore regions and all RI over the globe. RI was defined as (A) 45 kt/24 h and (B) 30 kt/24 h. The blue lines and dots show historical data. The orange lines show linear trends, with shading denoting a 95% confidence interval.

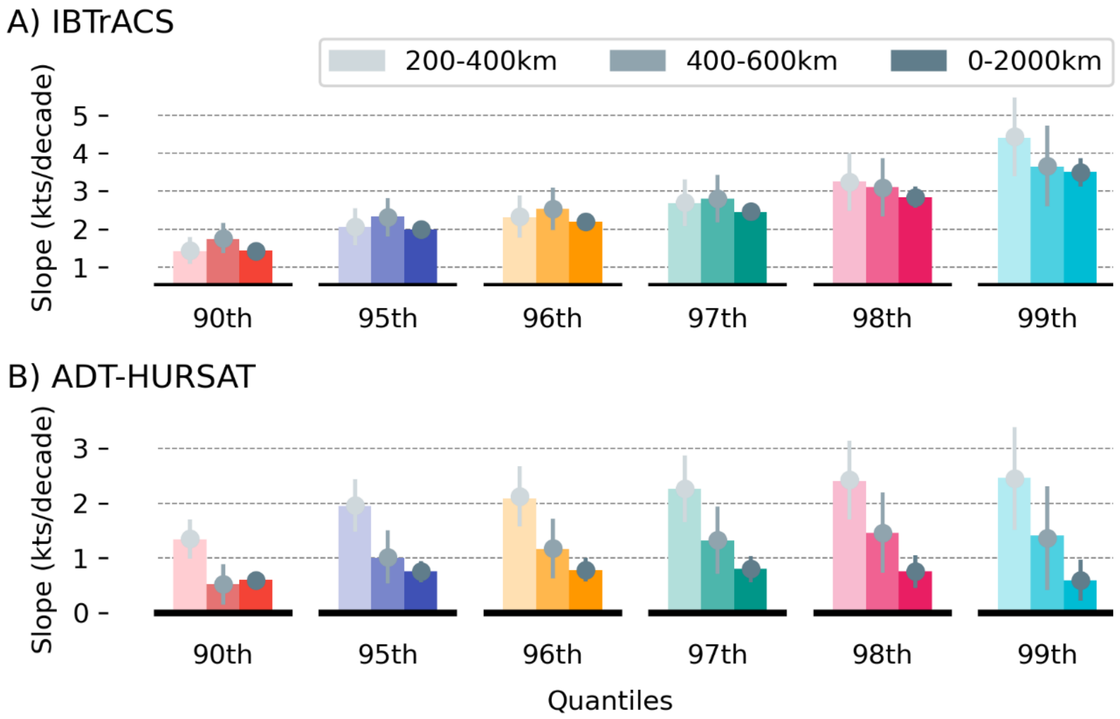

Supplementary Figure 2. Quantile regression of 24-h intensity changes. Slope of the quantiles for 24-h intensity changes for (A) IBTrACS during the period 1980–2020, and (B) ADT-HURSAT during the period 1982-2018. The error bar show the 5th and 95th percentiles of the regressions with randomly perturbed observational data.

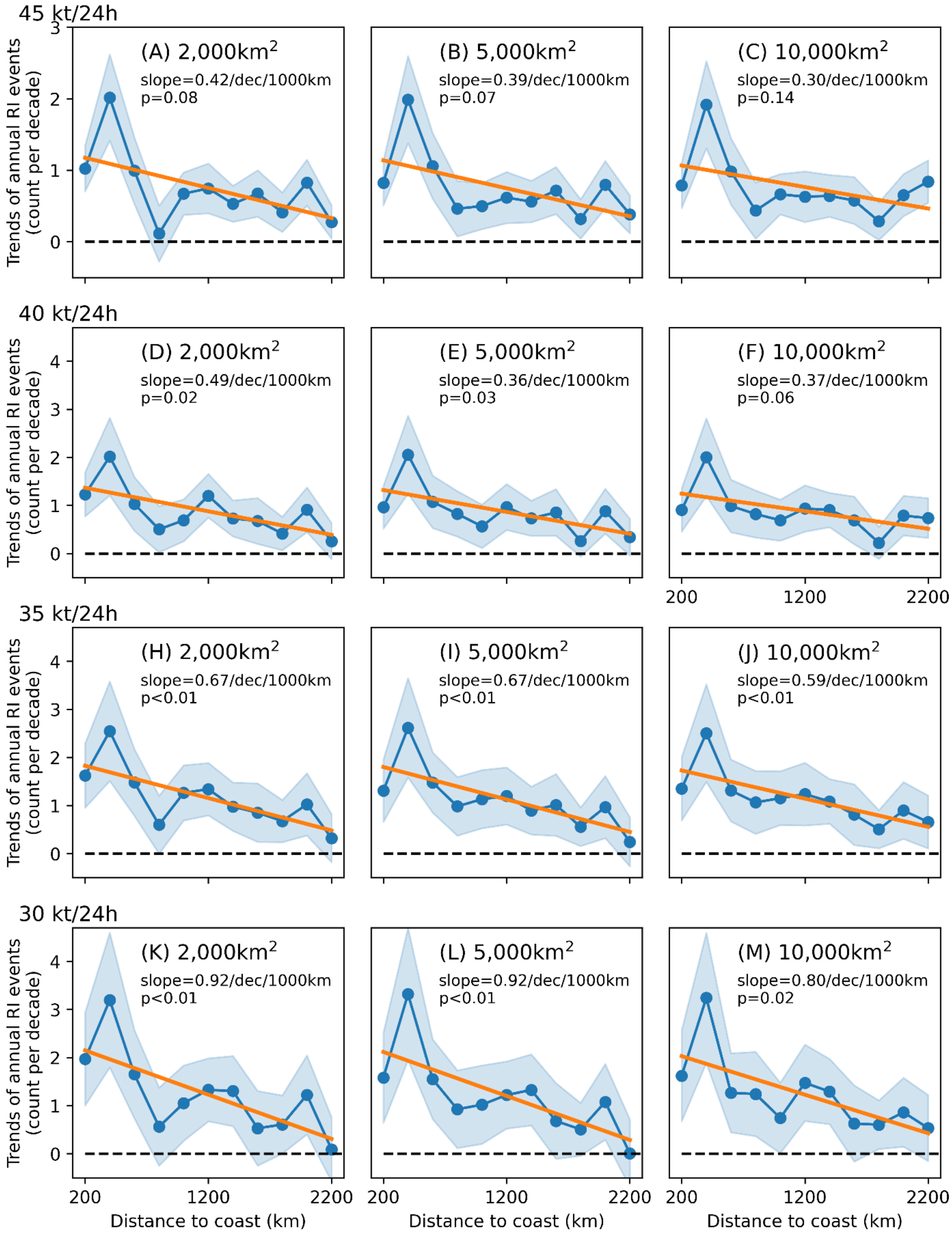

Supplementary Figure 3. Trends of annual RI counts with different distance to land, with the minimum landmass size of (left panel) 2000 km^2, (middle panel) 5000 km^2, and (right panel) 10000 km^2. RI is defined as an intensification of at least 45 kt/24 h for (A), (B) and (C); 40 kt/24 h for (D), (E) and (F); 40 kt/24 h for (H), (I) and (J); and 30 kt/24 h for (K), (L) and (M). The blue line and dots show linear temporal trends of the annual RI count for each 200-km bin, whereas the blue shading shows the 95% confidence level of the trends. The units are count/decade for the blue line and dots. The orange lines show linear fits of the temporal trends as a function of distance-to-land, and the units are count/decade/1,000 km. The slopes and p-values of the orange lines are shown on top of each subplot. The x-axis is the distance to land from

0–200 km to 2,000–2,200 km, with a 200-km interval. The y-axis is the temporal trend of the annual count of RI events within each 200-km bin.

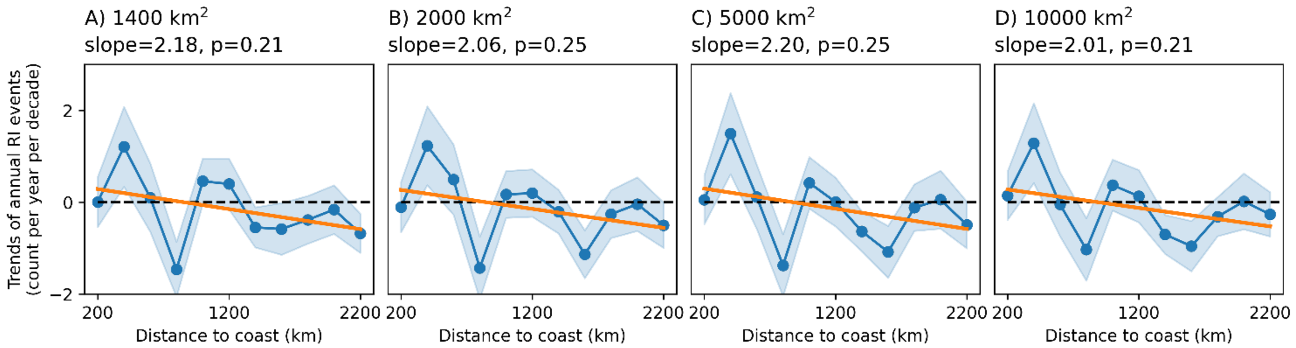

Supplementary Figure 4. Trends of annual RI counts in ADT-HURSAT dataset with different distance to land, with the minimum landmass size of (A) 1400 km^2, (B) 2000 km^2, (C) 5000 km^2, and (D) 10000 km^2. The data is for the period of 1982-2018. The RI is defined as an intensification of at least 45 kt/24 h. The blue line and dots show linear temporal trends of the annual RI count for each 200-km bin, whereas the blue shading shows the 95% confidence level of the trends. The units are count/decade for the blue line and dots. The orange lines show linear fits of the temporal trends as a function of distance-to-land, and the units are count/decade/1,000 km. The slopes and p-values of the orange lines are shown on top of each subplot. The x-axis is the distance to land from 0–200 km to 2,000–2,200 km, with a 200-km interval. The y-axis is the temporal trend of the annual count of RI events within each 200-km bin.

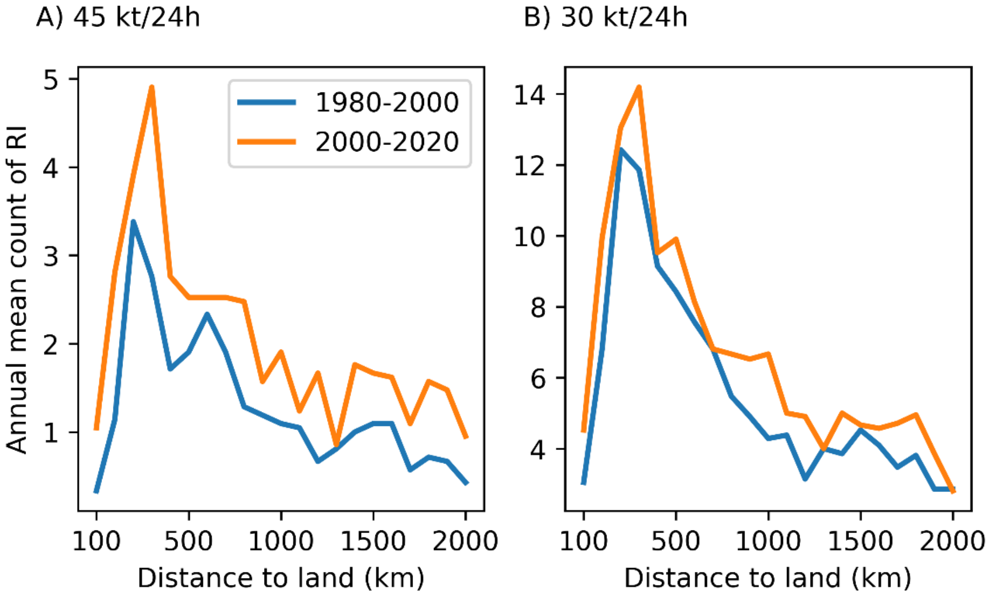

Supplementary Figure 5. Annual mean count of RI during 1980-2000 and 2000-2020. RI is defined as an intensification of at least (A) 45 kt/24 h and (B) 30 kt/24 h**.**

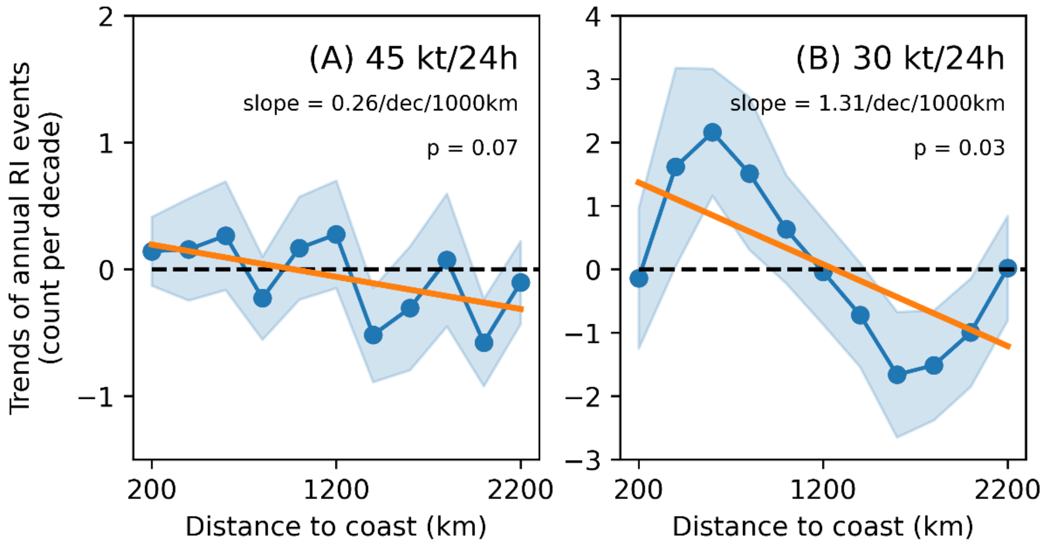

Supplementary Figure 6. Trends of annual RI counts from 1951 to 1979 with different distance to land. RI was defined as an intensification of at least (A) 45 kt/24h and (B) 30 kt/24h. The blue line and dots show linear temporal trends of the annual RI count for each 200-km bin, whereas the blue shading shows the 95% confidence level of the trends. The units are count/decade for the blue line and dots. The orange lines show linear fits of the temporal trends as a function of distance-to-land, and the units are count/decade/1,000 km. The slopes and p-values of the orange lines are shown on top of each subplot. The x-axis is the distance to land from 0–200 km to 2,000–2,200 km, with a 200-km interval. The y-axis is the temporal trend of the annual count of RI events within each 200-km bin.

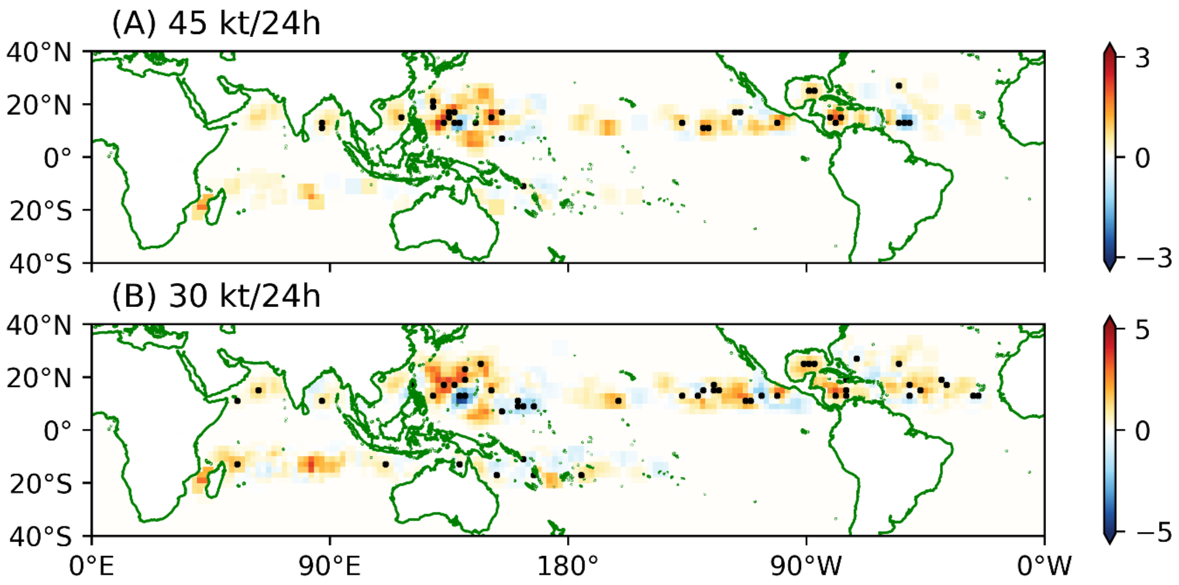

Supplementary Figure 7. Spatial distribution of the linear trend of annual RI ratios. RI was defined as an intensification of at least (A) 45 kt/24h and (B) 30 kt/24 h. The ratios were calculated as RI counts over TC activity for each 2 by 2 latitude-longitude grid. The 41-year linear trend is calculated for each 2° × 2° latitude–longitude grid, and the unit is %/decade. The black dots show areas where 95% confidence for the linear fit is satisfied. Data smoothing using a three-point smoother was performed for better display clarity.

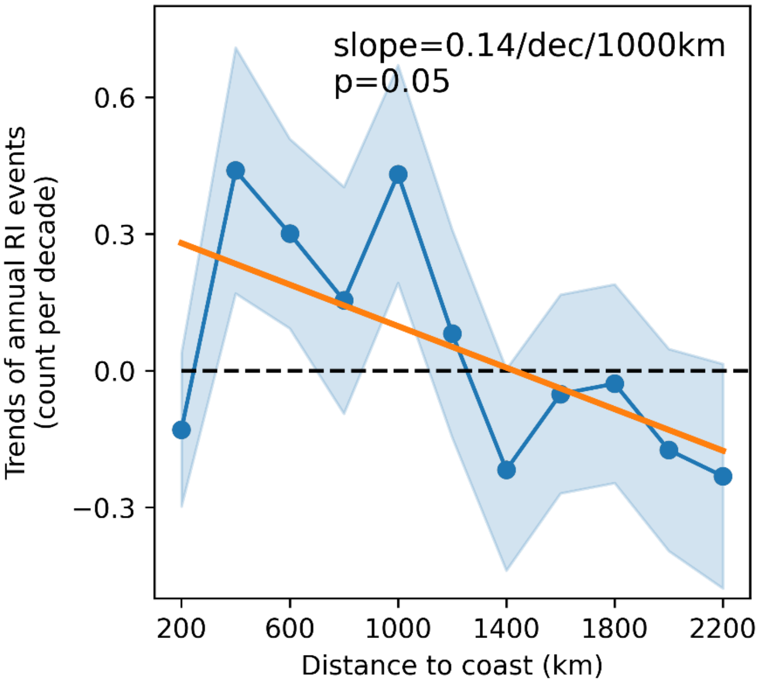

Supplementary Figure 8. Trends of annual RI counts with different distance to land for the western North Pacific, but with the mid-western North Pacific (130°E-150°E, 10°N-20°N) excluded. RI was defined as an intensification of at least 45 kt/24h. The x-axis is the distance to land from 0–200 km to 2,000–2,200 km, with a 200–km interval. The blue lines and shadings show linear temporal trends and a 95% confidence level of the trends, respectively. The orange lines show linear fits of the temporal trends as a function of distance to land.

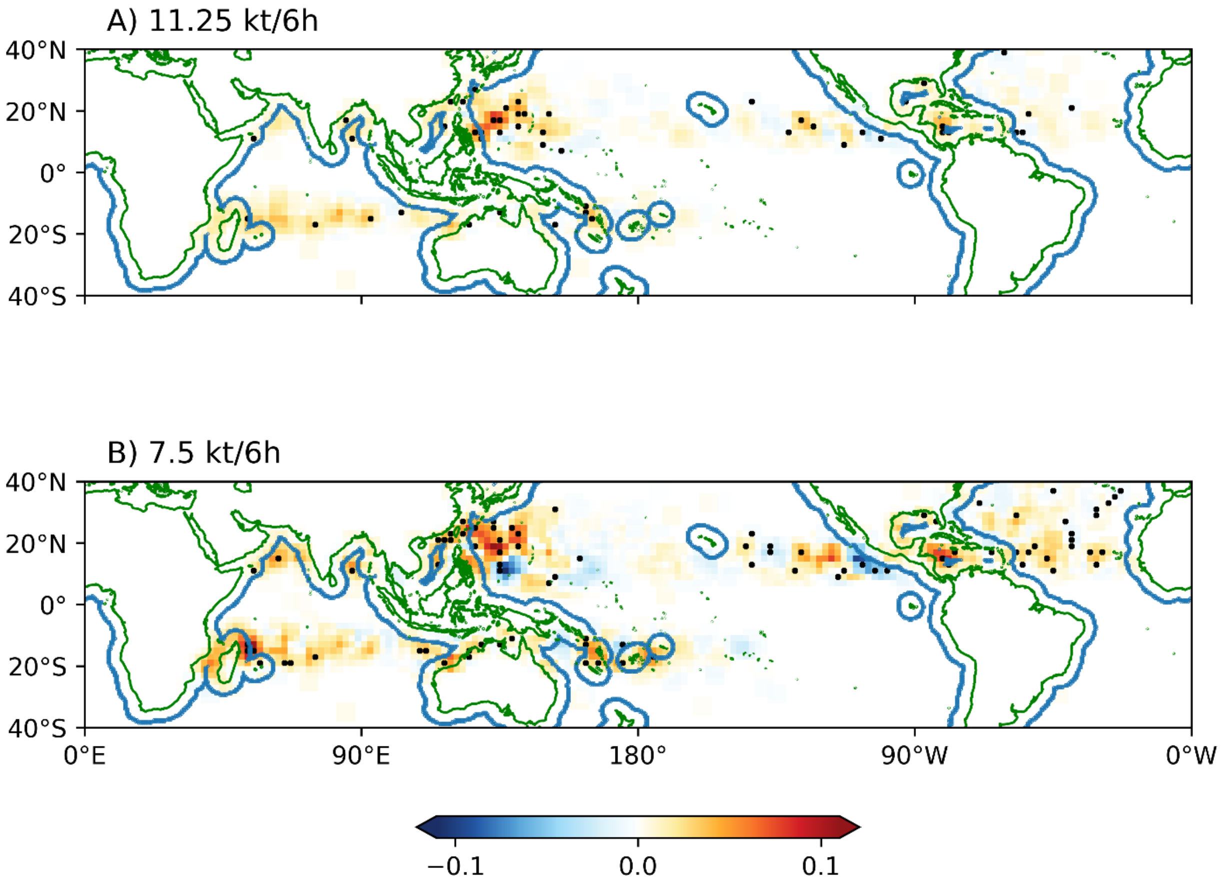

Supplementary Figure 9. Spatial distribution of the increasing trends of RI events. RI was defined as an intensification of at least (A) 11.25 kt/6h and (B) 7.5 kt/6h. The 41-year linear trend is calculated for each 2° × 2° latitude–longitude grid, and the unit is count/decade. The black dots show areas where 95% confidence for the linear fit is satisfied. The blue lines encompass the regions within 400 km from the coast. Data smoothing using a three-point smoother was performed for better display clarity.

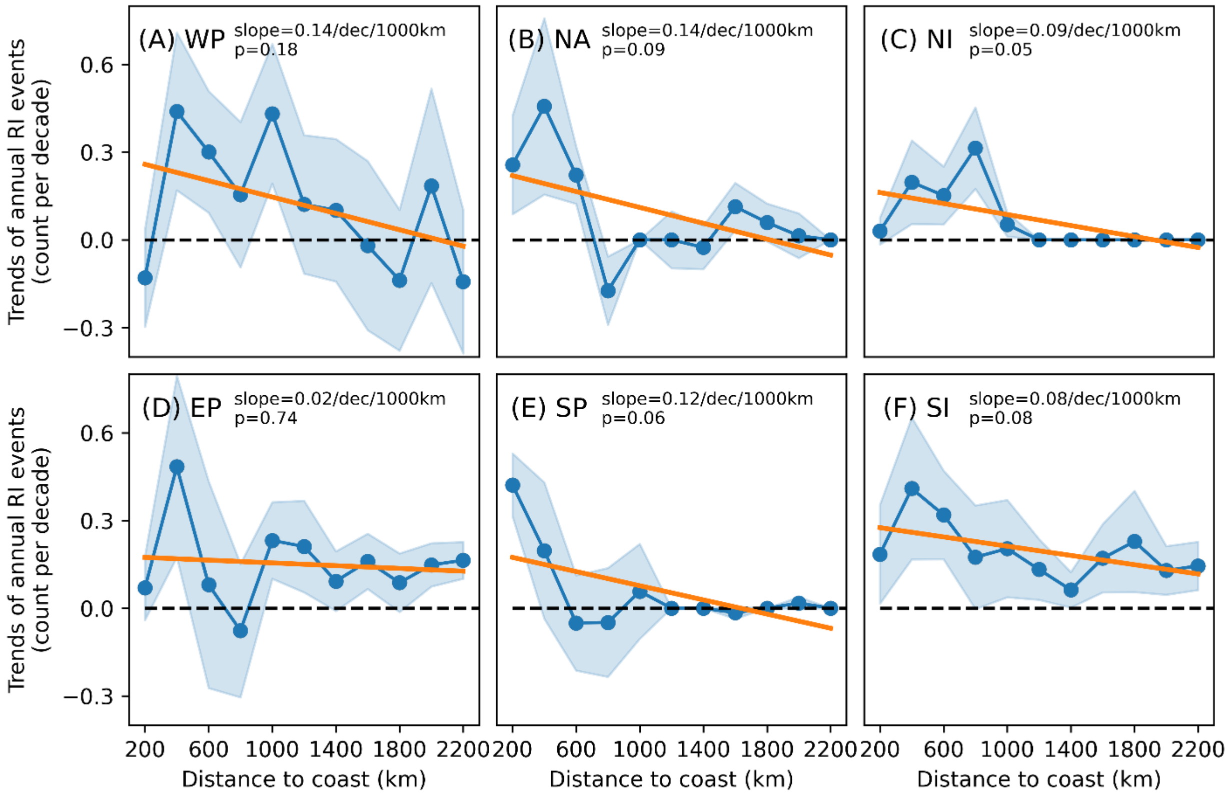

Supplementary Figure 10. Trends of annual RI counts with different distance to land for the (A) western North Pacific (WP), (B) North Atlantic (NA), (C) North Indian Ocean (NI), (D) eastern North Pacific (EP), (E) South Pacific (SP), and (F) South Indian Ocean. RI was defined as an intensification of at least 45 kt/24h. The x-axis is the distance to land from 0–200 km to 2,000–2,200 km, with a 200–km interval. The blue lines and shadings show linear temporal trends and a 95% confidence level of the trends, respectively. The orange lines show linear fits of the temporal trends as a function of distance to land.

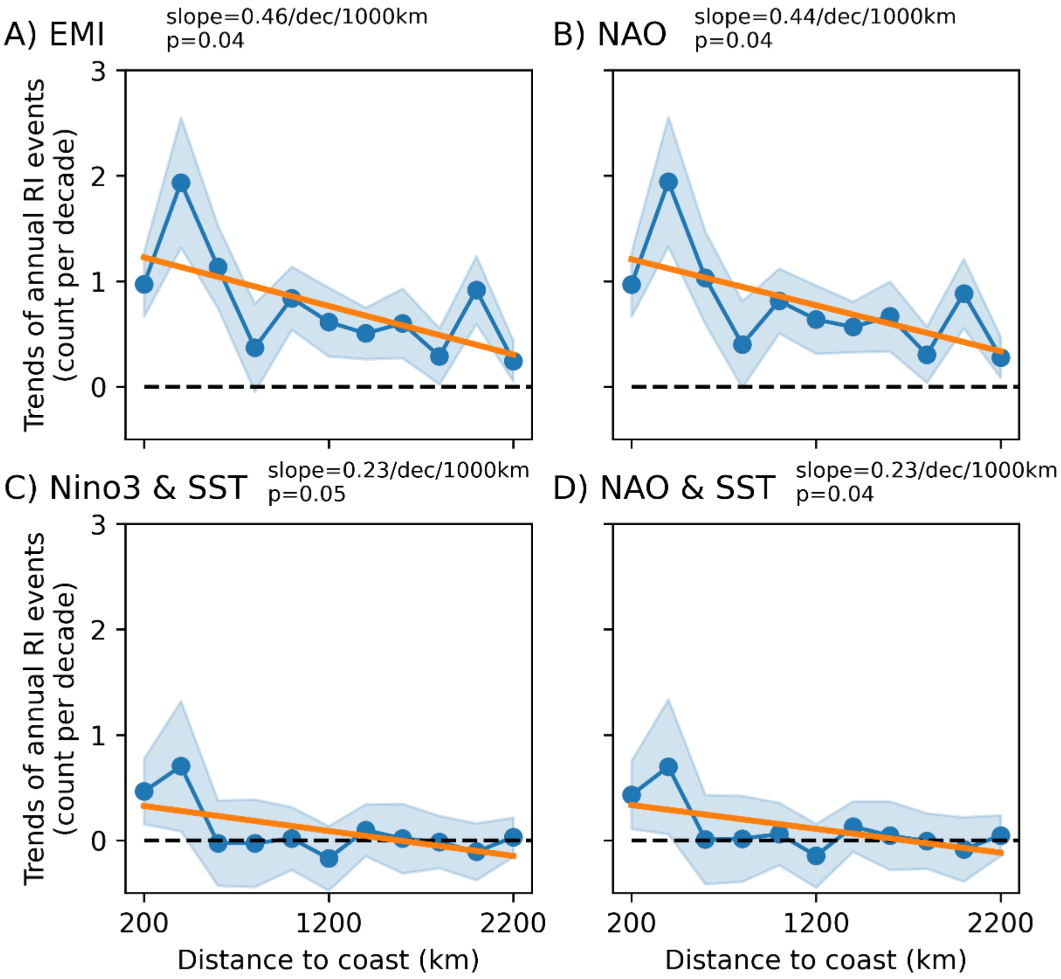

Supplementary Figure 11. Trends of RI events with climate indices and/or global SST trend reduced. A-D The effect of **(A)** Niño3 index, **(B)** PDO index, **(C)** global SST trend, and **(D)** both PDO and global SST were linearly reduced from the trend of the annual count of RI events. RI was defined as an intensification of at least 45 kt/24h. The x-axis is the distance to land from 0–200 km to 2,000–2,200 km, with a 200–km interval. The blue lines and shadings show linear temporal trends and a 95% confidence level of the trends, respectively. The orange lines show linear fits of the temporal trends as a function of distance to land.

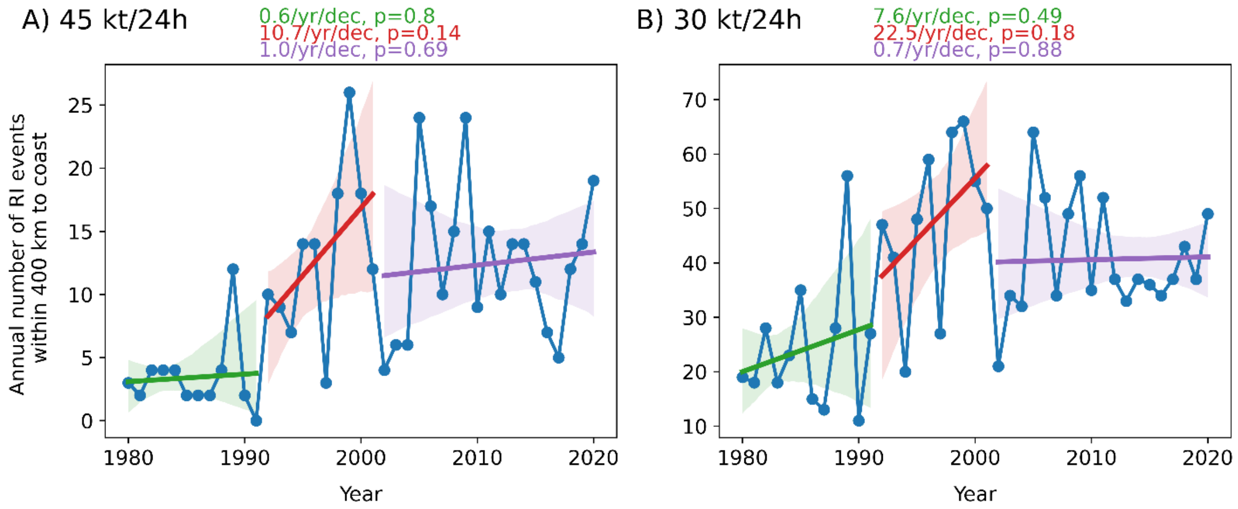

Supplementary Figure 12. Time series of annual mean count of RI events in the coastal regions. RI is defined as an intensification of at least A) 45 kt/24 h and B) 30 kt/24h. The blue lines and dots show historical data. The green, red and purple lines and shading show trends and uncertainties of the trends during 3 regimes (1980-1992,1992-2001,2002-2020). 2 regime shifts were detected at 1992 and 2002. The slopes and *p*-values were labeled for each regimes in the subplots.

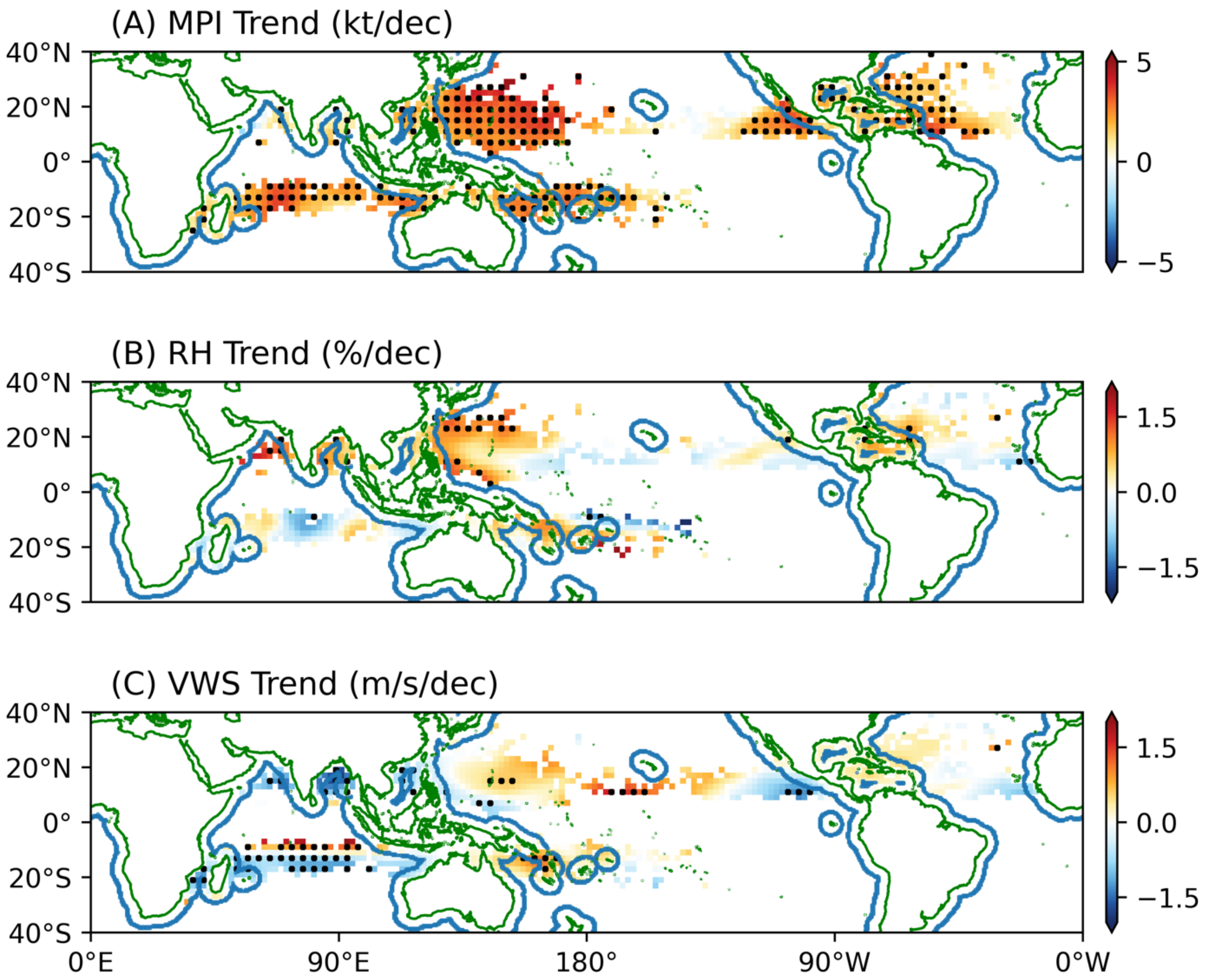

Supplementary Figure 13. Spatial distribution of linear trend of ambient conditions, including (A) maximum potential intensity (MPI), (B) 600-hPa relative humidity (RH), and (C) vertical wind shear (VWS) between 200- and 850-hPa. The trend is estimated from ERA5 data. The Black dots show areas where 95% confidence for the linear fit was satisfied. The blue lines encompass the regions within 400 km from the coast. The trends were calculated for each 2 by 2 longitude-latitude grid, and data smoothing using a three-point smoother was performed for better display clarity.

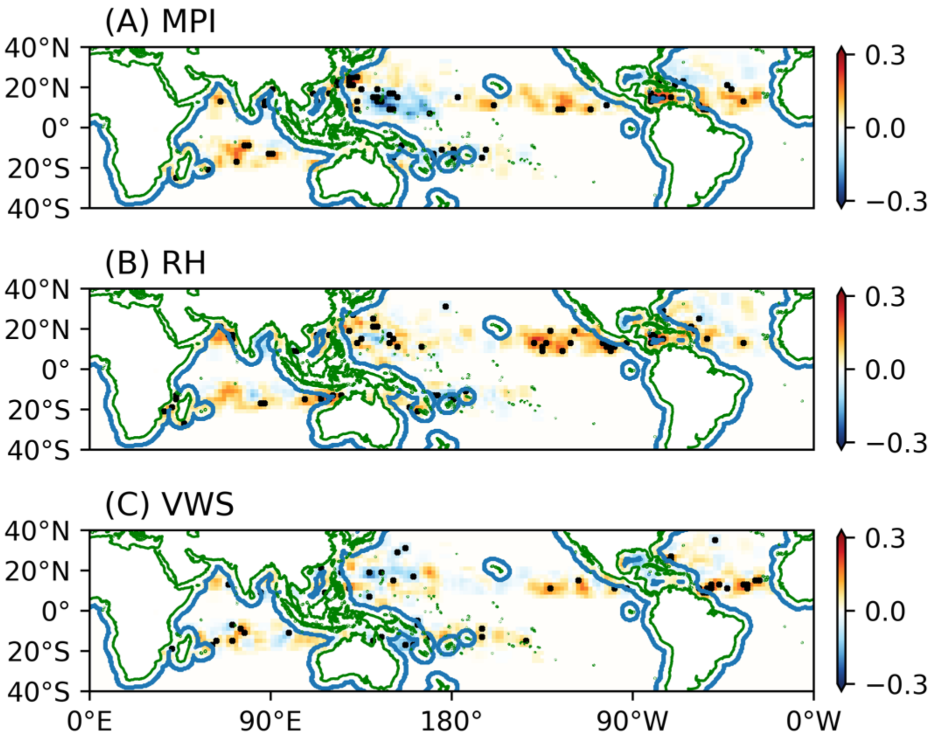

Supplementary Figure 14. Spatial distribution of correlation between annual mean count of RI and environment variables, including (A) maximum potential intensity (MPI), (B) 600-hPa relative humidity (RH), and (C) vertical wind shear (VWS) between 200- and 850-hPa. The correlation is estimated from ERA5. RI is defined as an intensification of at least 30 kt/24h for more events and robust analysis. Black dots show areas where 95% confidence for the linear fit was satisfied. The blue lines encompass the regions within 400 km from the coast. The correlation was calculated for each 2 by 2 longitude-latitude grid, and data smoothing using a three-point smoother was performed for better display clarity.

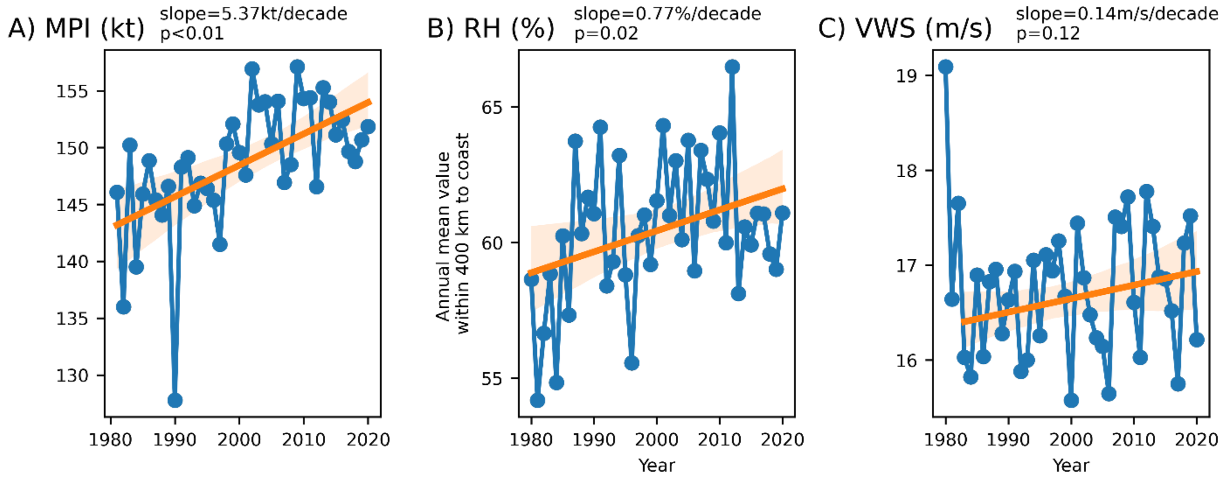

Supplementary Figure 15. Trends of annual mean (A) maximum potential intensity (MPI), (B) relative humidity (RH) and (C) vertical wind shear (VWS) with 400 km from 1980 to 2020. The trends were calculated using the ERA5 reanalysis. MPI was averaged within 200 km from the TC center, while RH and VWS were averaged within 200-800 km from the TC center. The blue dots and lines show the historical data. The orange lines show linear trends, with shading denoting a 95% confidence interval, with the slopes and *p*-values labeled in each subplot. The slope and *p*-value were calculated for 1983-2020 for VWS, to avoid the anomalous peak between 1980 and 1982.

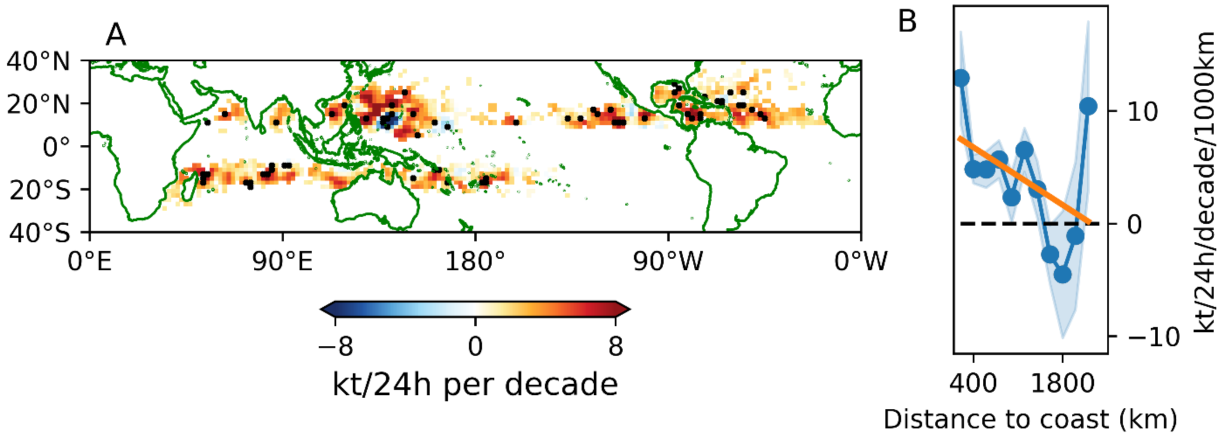

Supplementary Figure 16. Linear trend of potential intensification rate. A Spatial distribution of the linear trend of maximum potential intensification rate (MPIR, calculated from MPI). **B** Linear trend of MPIR with different distances-to-land. The trends were calculated using ERA5 reanalysis. In A, Black dots show areas where 95% confidence for the linear fit was satisfied. Data smoothing using a three-point smoother was performed for better display clarity in A. In B, the blue line and dots show linear temporal trends of PI values for each 200-km bin, while the blue shading shows the 95% confidence level of the trends. The orange lines show linear fits of the temporal trends as a function of distance-to-land, and the slope is 3.7 kt/24 h/decade/1,000 km, with $p<0.01$.

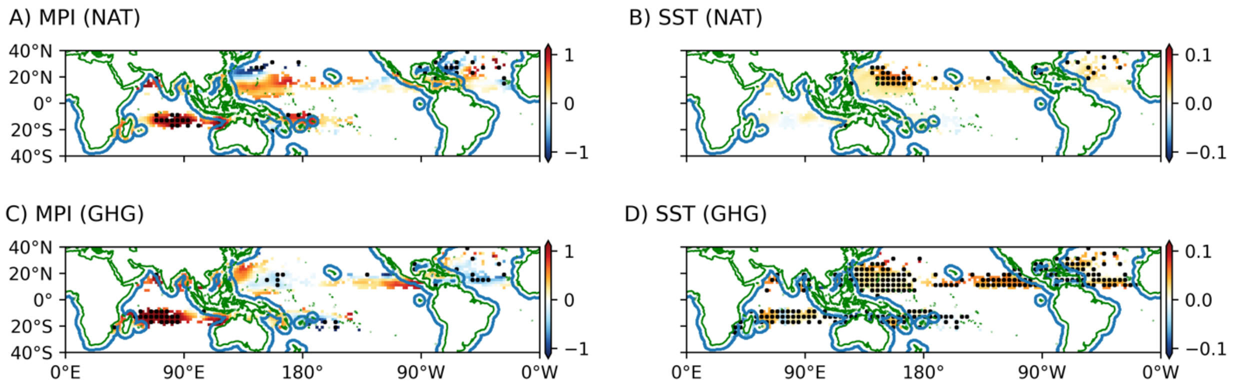

Supplementary Figure 17. Spatial distribution of the linear trend of MPI and SST estimated using the mean of (A) and (B) NAT and (C) and (D) GHG simulations in CMIP6. The black dots show areas where the 95% confidence interval of the linear fit was satisfied. The blue lines encompass the regions within 400 km from the coast. The trends were calculated for each 2 by 2 longitude-latitude grid, and data smoothing using a three-point smoother was performed for better display clarity.

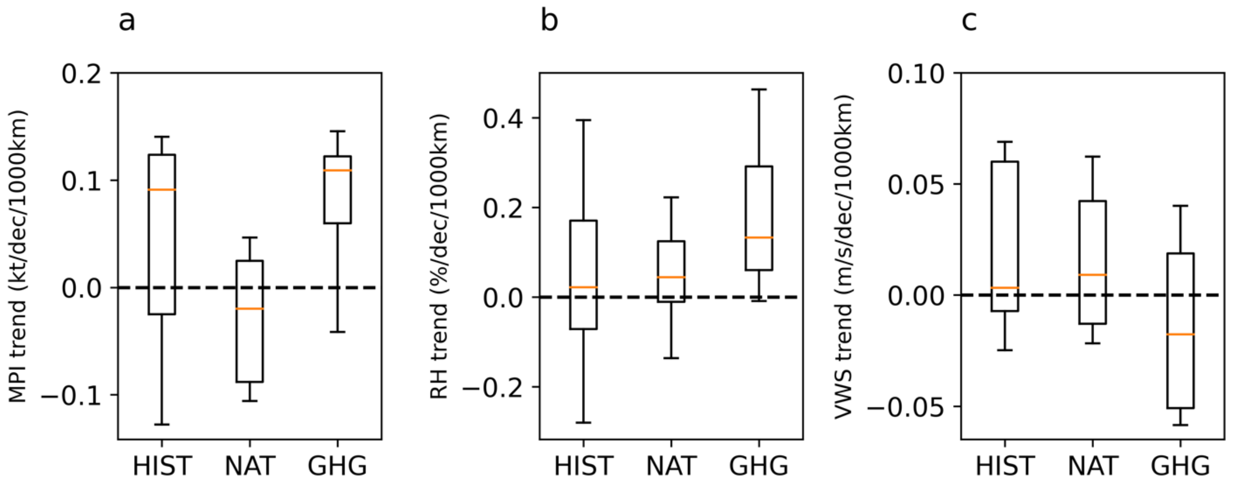

Supplementary Figure 18. Distribution of trend of the (a) MPI, (b) RH, and (c) VWS simulated in CMIP6 models over the period of 1950–2014. The models were forced with all forcing (HIST), natural variability (NAT), and anthropogenic forcing (greenhouse gas, GHG). A positive trend in the MPI and RH or a negative trend in the VWS indicates a more favorable environment in the offshore regions. The lower and upper ends of the box show the 25th and 75th quartiles, the middle line shows the median, and the horizontal bars below and above the box show the 5th and 95th quartiles, respectively.

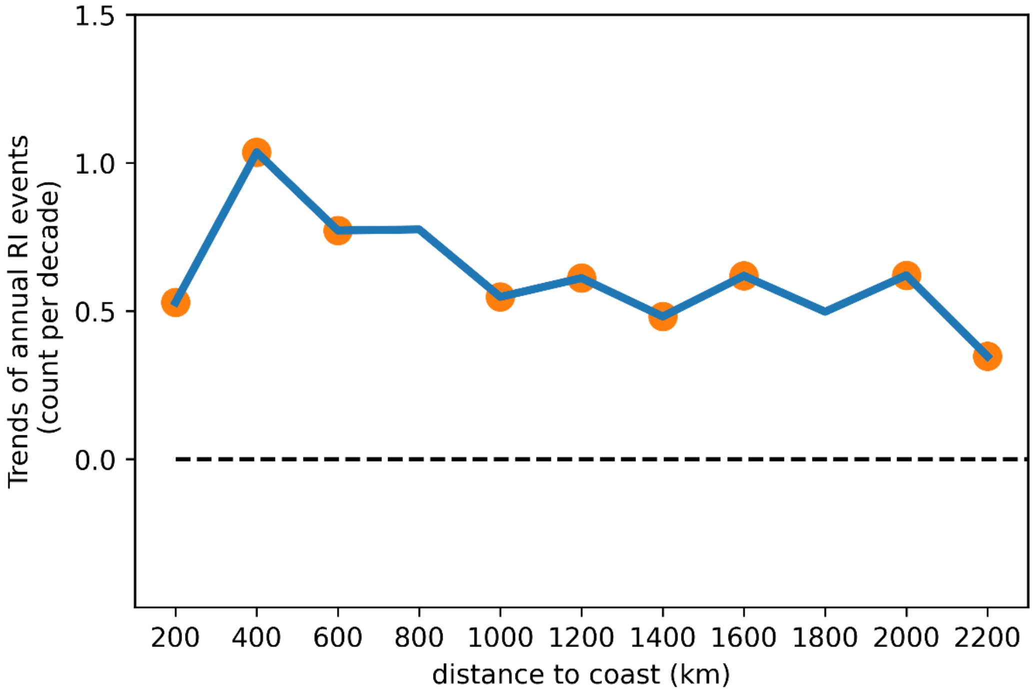

Supplementary Figure 19. Trends of annual RI counts with different distance to land, calculated using the improved ordinary least square algorithm[1]. RI is defined as an intensification of at least 45 kt/24 h. The x-axis is the distance to land from 0–200 km to 2000–2200 km, with a 200-km interval. The blue lines show linear temporal trends. The orange dots show the points where the trends are significant.

Supplementary References

1. Lian, T. Uncertainty in detecting trend: A new criterion and its applications to global SST. *Clim Dyn* **49**, 2881–2893 (2017).
